# The discovery of dendritic spines by Cajal

**DOI:** 10.3389/fnana.2015.00018

**Published:** 2015-04-21

**Authors:** Rafael Yuste

**Affiliations:** Department of Biological Sciences and Neuroscience, Neurotechnology Center, Columbia UniversityNew York, NY, USA

**Keywords:** dendritic spines, Cajal, Golgi, cortex, cerebellum

## Abstract

Dendritic spines were considered an artifact of the Golgi method until a brash Spanish histologist, Santiago Ramón y Cajal, bet his scientific career arguing that they were indeed real, correctly deducing their key role in mediating synaptic connectivity. This article reviews the historical context of the discovery of spines and the reasons behind Cajal's obsession with them, all the way till his deathbed.

Our story starts the spring of 1888, in Barcelona. At that time, this progressive and international city was undergoing a febrile creative period in literature, arts, architecture and industrial development, taking the leadership in the creation of modern Spain. A similar revolution was occurring in the relative quiet and obscurity of the Department of Histology of its Medical School, in the laboratory of Santiago Ramón y Cajal, a newly arrived Professor of Histology and Pathological Anatomy, who was starting his scientific career after a relatively tumultuous youth.

One of Cajal's personality traits was his strength of character. Indeed, Cajal was Aragonese and, in the popular culture of Spain, Aragonese and other northern Spaniards are considered to be single-minded and persistent. This is captured in a tale of an Aragonese farmer (“baturro”) riding his donkey on the train tracks and, when faced with an incoming train at full speed and blowing its whistle to warn him, tells the train that “blow as much as you want, but you are the one who needs to step out of the tracks.” Single-mindedness and persistence were combined in Cajal with a superb intuition and observation capabilities. Cajal himself credited his scientific successes not to his intelligence, education or training, but instead to his “will power,” combined with good experimental techniques, laboriousness and plain common sense (Ramón y Cajal, 1923).

On May 1st, precisely on the day of his 36th birthday, Cajal published a monograph entitled “Estructura de los centros nerviosos de las aves” (Structure of the Nervous Centers in Birds) in the first issue of a journal that he himself produced, edited, and financed (Ramón y Cajal, 1888). As he later wrote, the publication of this journal used up all his savings and prevented him and his wife from affording household help to care for their five children (Ramón y Cajal, 1923). In his monograph, a brief communication with two figures, Cajal described the application the Golgi stain to the cerebellum of birds. Cajal had just been taught the Golgi staining method by his friend Simarro in Madrid, who himself had recently learnt it from Ranvier in Paris, one of the premier neuroanatomists of the time (Fernandez and Breathnach, 2001). The Golgi impregnation enabled, for the first time, the relatively complete staining of the dendritic trees of neurons and is even today still widely used for the morphological analysis of dendrites. To a greater extent than any of his peers, Cajal had been struck by the power of the Golgi technique, particularly when applied to the developing nervous system, to reveal neuronal morphologies. In this brief article, Cajal noted that the surfaces of Purkinje cells were covered with small protrusions, which he called “espinas,” (i.e., “spines,” as in the spines of a rose, or “thorns”). In his own words: “… *Also, the surface of the Purkinje cells dendrites appear ruffled with thorns or short spines, which in the terminal dendrites look like light protrusions. Early on we thought that these eminences were the result of a tumultuous precipitation of the silver; but the constancy of their existence and its presence even in preparations where the staining appears with great delicacy in the remaining elements, incline us to consider them as a normal disposition*.” (Ramón y Cajal, 1888; translation by the author) (Figures 1, 2).

**Figure 1:**
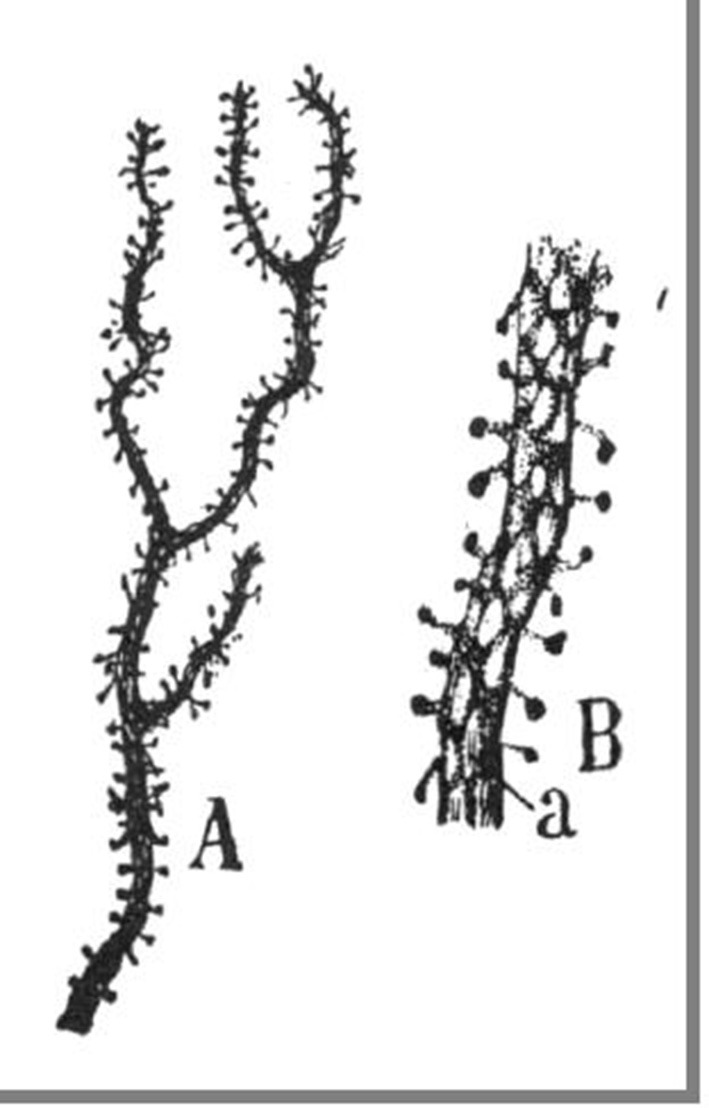
**Original illustrations from Cajal, displaying dendritic spines from a cerebellar Purkinje cell, as drawn from Golgi material (Ramón y Cajal, 1899c)**. Reproduced with permission from “Herederos de Santiago Ramón y Cajal.”

**Figure 2:**
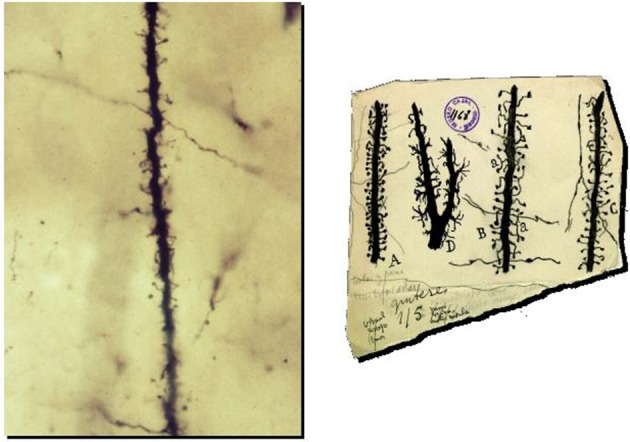
**Preparation and drawings of Cajal illustrating spines**. **Left:** Photomicrograph of a dendrite of pyramidal neuron from one of Cajal's original preparations (Courtesy of Cajal Institute in Madrid). **Right:** Cajal drawings of spines from rabbit (A), 2 month old child (B), one month old cat (C) and cat spinal motoneuron (D). Reproduced with permission from “Herederos de Santiago Ramón y Cajal.”

In this relatively brief communication, written in Spanish, a language not commonly used by international scientists, spines were described and named for the first time. In this same publication Cajal could not confirm the presence of anastomoses between axons and dendrites, hypothesized to exist by Golgi and other investigators, and proposed that neurons are independent units in the nervous system. This assertion, in agreement with ideas from other investigators (Bock, 2013), laid the basis of the “neuron doctrine,” an opposing hypothesis to Golgi's established “reticular theory,” in which neurons would form a continuous network of physically joined cells (see Shepherd, 1991). Thus, in the same publication, he changed the core of Neuroscience, with two fundamental and apparently unrelated observations: neurons are independent from each other and are covered with spines. In his later career he will proceed to link both facts into our modern conception of the brain.

To put this 1888 study in perspective, it should be noted that Cajal was not the first one to use the Golgi method and also not the first to observe spines. Other investigators, like Kolliker, Dogiel, Meyer and even Golgi himself, more established than Cajal and working in well-recognized centers of anatomical research at the time, had observed spines before him. However, these researchers regarded spines as fixation artifacts or silver precipitates outside the neurons, and in their scientific publications they drew neurons with smooth dendritic trees, devoid of spines. But even today, spines are still clearly visible in Camilo Golgi's original preparations (Purpura, pers. comm.), so Golgi must have to ignore them since he drew neurons with smooth surfaces. This was not such an unreasonable choice considering that the Golgi method is notoriously capricious and variable in results. Still today, it is poorly understood how exactly Golgi impregnations work. To make things more confusing, other observable structures on the surface of neurons, such as dendritic varicosities, were thought to be artifactual by Cajal himself (Ramón y Cajal, 1904). So it is understandable how Cajal's proposal that spines were real structures was met with skepticism.

Rather than buckle under the pressure of his contemporaries, and perhaps shielded from them due to his relative isolation, far from the centers of scientific inquiry of his time, Cajal pressed on in his studies on the structure of spines, in a flurry of publications that followed. Shortly afterwards, Cajal revealed that spines are not particular to birds but are also present in the dendrites of many neurons of the cerebral cortex of mammals (Ramón y Cajal, 1891b). Importantly, he speculated that spines must receive axonal inputs from other neurons, and thus serve as the main point of contact between axons and dendrites (Ramón y Cajal, 1894). This is the point where his neuronal doctrine came full circle: neurons are independent from each other but (at least those in the cerebral cortex) they connect to one another through their axons and spines.

Being curious and inquisitive, Cajal wondered what was the advantage of using spines as recipient sites for axonal connections, given that axons could in principle connect directly to the dendritic trunk. He proposed the idea that spines would greatly extend the surface of the dendrites, and therefore dramatically increase their capability to receive axons. This hypothesis was based on the comparison between spines and intestinal villi, where a highly branched structure increases the surface area of the cell. In addition, Cajal proposed that physical changes in spines could be associated with neuronal function and learning (Ramón y Cajal, 1891a, 1893). Imagining that his histological preparations were still alive, he argued that, in the living animal, spines could move and change, growing with activity and retracting during inactivity or sleep. So physical movements of the spines could be capable of connecting or disconnecting neurons. As he put it “*Since it seems rather likely that the named spines represent points of charge or of current gathering, their retraction (which in this fashion would isolate them from the terminal nerve fibers, with which they are in contact) would give rise to the individualization or separation of neurons*” (Ramón y Cajal, 1899c). Indeed, one of the most exciting recent findings has been the discovery that spines are not stable structures, but are constantly moving and experience morphological plasticity *in vivo* and *in vitro* (Figure 3) (Fischer et al., 1998; Dunaevsky et al., 1999). Therefore, although Cajal's intuition that spines can connect and disconnect during the day cycle has not yet been demonstrated, the general idea that spines are morphologically plastic is still central to the study of the function of spines.

**Figure 3:**
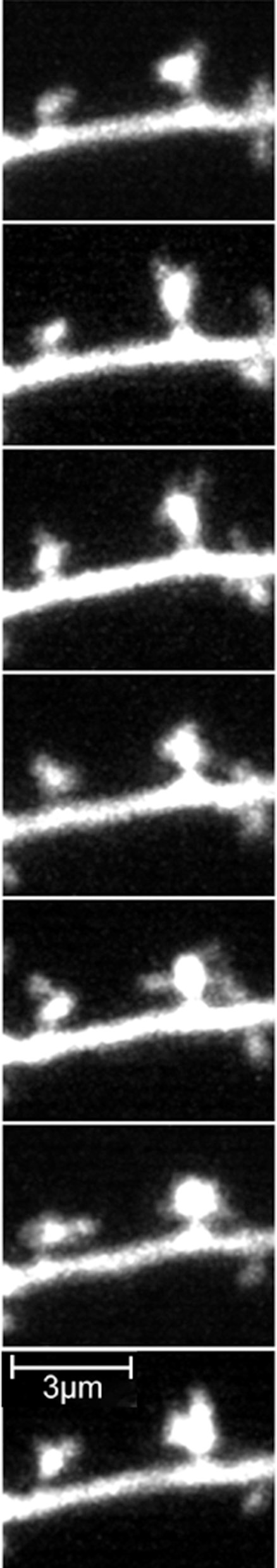
**Spine morphological plasticity in a pyramidal neuron**. Frames from a two-photon movie of 8.5 min duration of GFP labeled pyramidal neurons of postnatal cortical brain slice from a postnatal mouse. Note the large morphological plasticity of the spines. Reprinted with permission from (Portera-Cailliau et al., 2003).

In 1896, partly to defend himself from attacks that his so-called spines were artifacts of the Golgi method and did not appear with other staining procedures, Cajal extended his Golgi observations of spines using a different method, the Ehrlich methylene-blue stain (Ramón y Cajal, 1896a,b). In this publication, he refined this technique and showed that it could also reveal spine morphologies, when properly used.

In subsequent years, Cajal described with great detail spines in motor, visual, auditory and olfactory human cortices (Ramón y Cajal, 1899a,b, 1900a,b). In 1899, he summarized many of his observations on his book “Histology of the Nervous System of Man and Vertebrates,” where he restated his view that spines increase the surface area of dendrites and thus serve as site of contacts between dendrites and axons. In an additional effort to convince his colleagues, he collected together all his arguments that spines were not artifactual, because:

1. Spines are shown by different methods, like Golgi, Cox or methylene blue stains.
2. They always arise in the same position of the neuron, from the same regions of the brain.
3. Spines are never or rarely found in certain parts of the neuron (like the axon, soma or initial dendrites).
4. Spines do not resemble crystal deposits when viewed with higher power objectives.
5. Spine pedicles (necks) can be occasionally detected.
6. Spines can be stained by neurofibrillary methods.

Moreover, noting that cells from more highly evolved animals have more spines, he argued that spines were probably related to intelligence (Ramón y Cajal, 1899c, 1904).

Finally, in one of his last contributions to the problem, Cajal discussed which axons specifically contact spines (Ramón y Cajal, 1933). Cajal argues that spines can be contacted by different types of axons. According to him, in cortical pyramidal neurons, spines can be contacted by: (i) axonal collaterals from other pyramidal cells, (ii) axons from some interneurons (Golgi type II cells), and (iii) axons from other associative neurons.

Cajal was obsessed with spines, and he undertook a personal crusade, pretty much alone and till his deathbed, to convince his peers that spines were not only real, but also crucially important. Indeed, on his deathbed, Cajal was still arguing about spines. In a letter in shaky handwriting to his disciple Lorente de Nó on October 15th, 1934, 2 days before he died (Figure 4), after reporting that he is so sick that he cannot leave his bed or work anymore, he advises Lorente to pay close attention to spines. He writes: “….*Note that spines are not irregular protrusions but instead genuine spines ending in a ball. The neck is sometimes too lightly stained* … ” (Copy of autograph letter to Lorente, courtesy of Dr. Francisco Alvarez, Creighton University, translation by the author).

**Figure 4:**
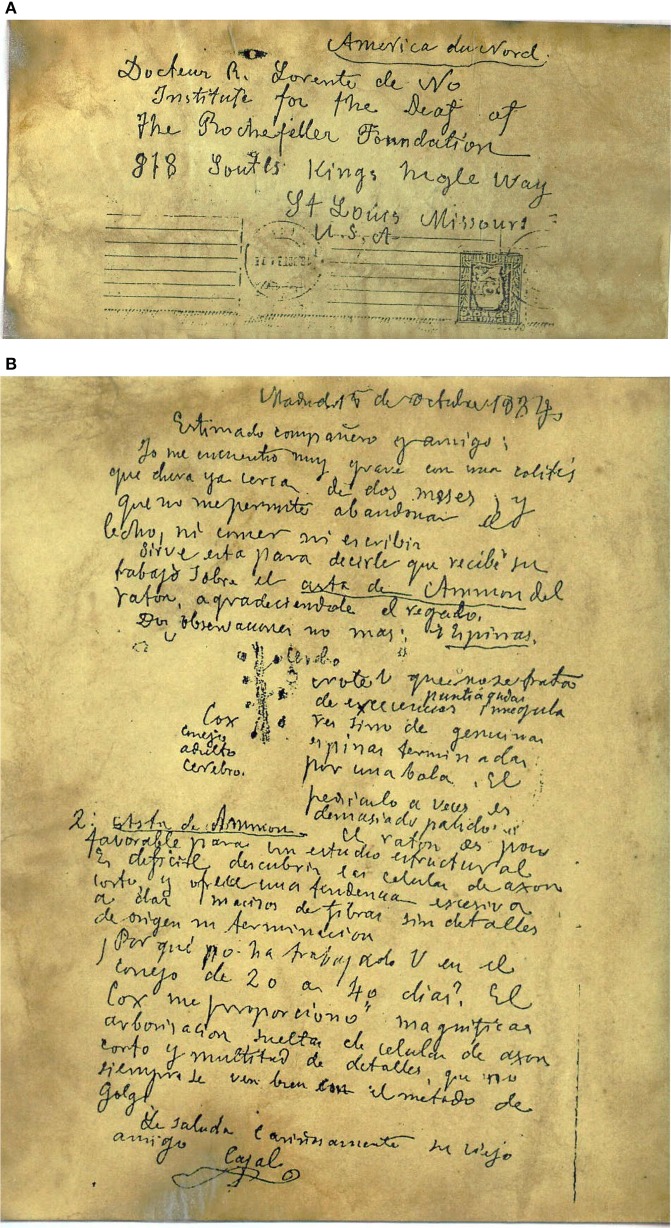
**Letter from Cajal to Lorente de Nó. (A)** Envelope addressed to R. Lorente de Nó, Institute of the Deaf at The Rockefeller Foundation in St. Louis, Missouri, USA. **(B)** Manuscript letter. Note the drawing of dendritic spines. Paragraph is translated in the text. Reproduced with permission from “Herederos de Santiago Ramón y Cajal.”

In spite of this string of arguments and the combined weight of his evidence, Cajal's conclusions were not readily accepted. Eventually, many of his contemporaries, such as Retzius, Schaffer, Edinger, Azolay, Berkley, Monti, and Stefanowska came to agree with him and confirm their appearance in their preparations.

At the same time, not much work was carried out on spines and Cajal's proposal of the role of spines in connecting axons and dendrites would have to wait till midcentury for its confirmation. This occurred by the introduction of a new technology, electron microscopy, which enabled the visualization of the fine structure of cells with unprecedented spatial resolution. Indeed, in the 1950s, De Robertis and Palay performed the first ultrastructural analysis of synapses (DeRobertis and Bennett, 1955; Palay, 1956) and shortly afterwards, synapses were demonstrated on spines (Gray, 1959a,b). Cajal was proven correct and spines became a bona-fide topic of interest for neurobiological studies.

Since the 1950's, each decade has brought along an increased number of studies of spines, with a recent acceleration of studies published since 1990. Nevertheless, the specific function of the spine, more than a hundred after their discovery, is still subject to great debate and many different hypotheses have been proposed (Peters and Kaiserman-Abramof, 1970; Swindale, 1981; Harris and Kater, 1994; Shepherd, 1996; Harris, 1999; Yuste et al., 2000; Yuste and Majewska, 2001; Alvarez and Sabatini, 2007; Bourne and Harris, 2008). Our knowledge of spine morphology, ultrastructure, biochemistry, development, dynamics, calcium compartmentalization, biophysical properties and electrophysiology, has exploded. This rich phenomenology has opened up many questions related to spines, indicating their importance. As Cajal wrote “the future will prove the great physiological role played by the spines” (Ramón y Cajal, 1904).

## Conflict of interest statement

Modified with permission from Yuste R. (2010), Dendritic Spines, MIT Press, Cambridge, Massachusetts. The author declares that the research was conducted in the absence of any commercial or financial relationships that could be construed as a potential conflict of interest.

## References

[B1] Alvarez V. Sabatini B. (2007). Anatomical and physiological plasticity of dendritic spines. Annu. Rev. Neurosci. 30, 79–97. 10.1146/annurev.neuro.30.051606.094222 17280523

[B2] Bock O. (2013). Cajal, Golgi, Nansen, Schafer and the neuron doctrine. Endeavour 37, 228–234. 10.1016/j.endeavour.2013.06.006 23870749

[B3] Bourne J. N. Harris K. M. (2008). Balancing structure and function at hippocampal dendritic spines. Annu. Rev. Neurosci. 31, 37–67. 10.1146/annurev.neuro.31.060407 18284372 PMC2561948

[B4] DeRobertis E. D. P. Bennett H. S. (1955). Some features of the submicroscopic morphology of synapses in frog and earthworm. J. Biophys. Biochem. Cytol. 1, 47–58. 10.1083/jcb.1.1.47 14381427 PMC2223594

[B5] Dunaevsky A. Tashiro A. Majewska A. Mason C. A. Yuste R. (1999). Developmental regulation of spine motility in mammalian CNS. Proc. Natl. Acad. Sci. U.S.A. 96, 13438–13443. 10.1073/pnas.96.23.13438 10557339 PMC23966

[B6] Fernandez N. Breathnach C. S. (2001). Luis Simarro Lacabra [1851-1921]: from Golgi to Cajal through Simarro, via Ranvier? J. Hist. Neurosci. 10, 19–26. 10.1076/jhin.10.1.19.5622 11446260

[B7] Fischer M. Kaech S. Knutti D. Matus A. (1998). Rapid actin-based plasticity in dendritic spine. Neuron 20, 847–854. 10.1016/S0896-6273(00)80467-5 9620690

[B8] Gray E. G. (1959a). Axo-somatic and axo-dendritic synapses of the cerebral cortex: an electron microscopic study. J. Anat. 83, 420–433. 13829103 PMC1244535

[B9] Gray E. G. (1959b). Electron microscopy of synaptic contacts on dendritic spines of the cerebral cortex. Nature 183, 1592–1594. 10.1038/1831592a0 13666826

[B10] Harris K. M. (1999). Structure, development, and plasticity of dendritic spines. Curr. Opin. Neurobiol. 9, 343–348. 10.1016/S0959-4388(99)80050-6 10395574

[B11] Harris K. M. Kater S. B. (1994). Dendritic spines: cellular specializations imparting both stability and flexibility to synaptic function. Annu. Rev. Neurosci. 17, 341–371. 10.1146/annurev.ne.17.030194.002013 8210179

[B12] Palay S. L. (1956). Synapses in the central nervous system. J. Biophysiol. Biochem. Cytol. 2, 193–201 10.1083/jcb.2.4.193 PMC2229686 13357542

[B13] Peters A. Kaiserman-Abramof I. R. (1970). The small pyramidal neuron of the rat cerebral cortex. The perykarion, dendrites and spines. J. Anat. 127, 321–356. 10.1002/aja.1001270402 4985058

[B13a] Portera-Cailliau C. Pan D. Yuste R. (2003). Activity-regulated motility of early dendritic protrusions: evidence for different types of dendritic filopodia. J. Neurosci. 23, 7129–7142. 12904473 10.1523/JNEUROSCI.23-18-07129.2003 PMC6740658

[B14] Ramón y Cajal S. (1888). Estructura de los centros nerviosos de las aves. Rev. Trim. Histol. Norm. Pat. 1, 1–10.

[B15] Ramón y Cajal S. (1891a). Significación fisiológica de las expansiones protoplásmicas y nerviosas de la sustancia gris. Revista de Ciencias Médicas de Barcelona 22, 23.

[B16] Ramón y Cajal S. (1891b). Sur la structure de l'ecorce cerebrale de quelques mamiferes. La Cellule 7, 124–176.

[B17] Ramón y Cajal S. (1893). Neue darstellung vom histologischen bau des centralnervensystem. Arch. Anat. Entwick. 1893, 319–428.

[B18] Ramón y Cajal S. (1894). La fine structure des centres nerveux. The croonian lecture. Proc. R. Soc. Lond. B Biol. Sci. 55, 443–468.

[B19] Ramón y Cajal S. (1896a). Le bleu de methylene dans les centres nerveaux. Rev. Trim. Microgr. 1, 21–82.

[B20] Ramón y Cajal S. (1896b). Les epines collaterales des cellules du cerveau colorees au bleu de methylene. Rev. Trim. Microgr. 1, 5–19.

[B21] Ramón y Cajal S. (1899a). Estudios sobre la cortexa cerebral humana. Corteza visual. Rev. Trim. Microgr. 4, 1–63.

[B22] Ramón y Cajal S. (1899b). Estudios sobre la cortexa cerebral humana. Estructura de la cortex motriz del hombre y mamiferos. Rev. Trim. Microgr. 4, 117–200.

[B23] Ramón y Cajal S. (1899c). La Textura del Sistema Nerviosa del Hombre y los Vertebrados. Madrid: Moya (Primera Edicion).

[B24] Ramón y Cajal S. (1900a). Estudios sobre la cortexa cerebral humana. Esctructura de la corteza acustica. Rev. Trim. Microgr. 5, 129–183.

[B25] Ramón y Cajal S. (1900b). Estudios sobre la cortexa cerebral humana. Estructura de la corteza cerebral olfativa del hombre y mamiferos. Rev. Trim. Microgr. 6, 1–150.

[B26] Ramón y Cajal S. (1904). La Textura del Sistema Nerviosa del Hombre y los Vertebrados. Moya: Madrid.

[B27] Ramón y Cajal S. (1923). Recuerdos de mi Vida: Historia de mi Labor Científica. Madrid: Alianza Editorial.

[B28] Ramón y Cajal S. (1933). Neuronismo o reticularismo? Las Pruebas Objetivas de la Unidad Anatomica de las Celulas Nerviosas. Madrid: Instituto Cajal.

[B29] Shepherd G. (1996). The dendritic spine: a multifunctional integrative unit. J. Neurophysiol. 75, 2197–2210. 8793734 10.1152/jn.1996.75.6.2197

[B30] Shepherd G. M. (1991). Foundations of the Neuron Doctrine. Oxford: Oxford University Press.

[B31] Swindale N. V. (1981). Dendritic spines only connect. Trends Neurosci. 4, 240–241. 10.1016/0166-2236(81)90075-811597098

[B32] Yuste R. Majewska A. (2001). On the function of dendritic spines. Neuroscientist 7, 387–395. 10.1177/107385840100700508 11597098

[B33] Yuste R. Majewska A. Holthoff K. (2000). From form to function: calcium compartmentalization in dendritic spines. Nat. Neurosci. 3, 653–659. 10.1038/76609 10862697

